# Cancer-epigenetic function of the histone methyltransferase KMT2D and therapeutic opportunities for the treatment of KMT2D-deficient tumors

**DOI:** 10.18632/oncotarget.27988

**Published:** 2021-06-22

**Authors:** Shilpa S. Dhar, Min Gyu Lee

**Affiliations:** ^1^Department of Molecular and Cellular Oncology, The University of Texas MD Anderson Cancer Center, Houston, TX 77030, USA; ^2^The Center for Cancer Epigenetics, The University of Texas MD Anderson Cancer Center, Houston, TX 77030, USA

**Keywords:** cancer, epigenetics, histone methyltransferase, KMT2D, MLL4

## Abstract

Epigenetic mechanisms are central to understanding the molecular basis underlying tumorigenesis. Aberrations in epigenetic modifiers alter epigenomic landscapes and play a critical role in tumorigenesis. Notably, the histone lysine methyltransferase KMT2D (a COMPASS/ Set1 family member; also known as MLL4, ALR, and MLL2) is among the most frequently mutated genes in many different types of cancer. Recent studies have demonstrated how KMT2D loss induces abnormal epigenomic reprograming and rewires molecular pathways during tumorigenesis. These findings also have clinical and therapeutic implications for cancer treatment. In this review, we summarize recent advances in understanding the role of KMT2D in regulating tumorigenesis and discuss therapeutic opportunities for the treatment of KMT2D-deficient tumors.

## INTRODUCTION

Covalent modifications of histones sophisticatedly regulate chromatin architecture [1–4]. Of the histone modifications, histone lysine (K) methylation is crucial to the epigenetic and transcriptional regulation of gene expression. This modification is associated with either activation or silencing of gene expression, and its effect on gene expression is dependent on methylated lysine sites. For instance, methylations at histone H3 lysine 4 (H3K4), H3K36, and H3K79 are generally considered activation marks (or signals), whereas methylations at H3K9, H3K27, and H4K20 are known to be repressive marks. Lysine methylation can take place at three different levels (mono-, di- or trimethyl) at specific lysine residues within histones and is catalyzed by lysine methyltransferases (KMTs) [4, 5].

**Figure 1:**
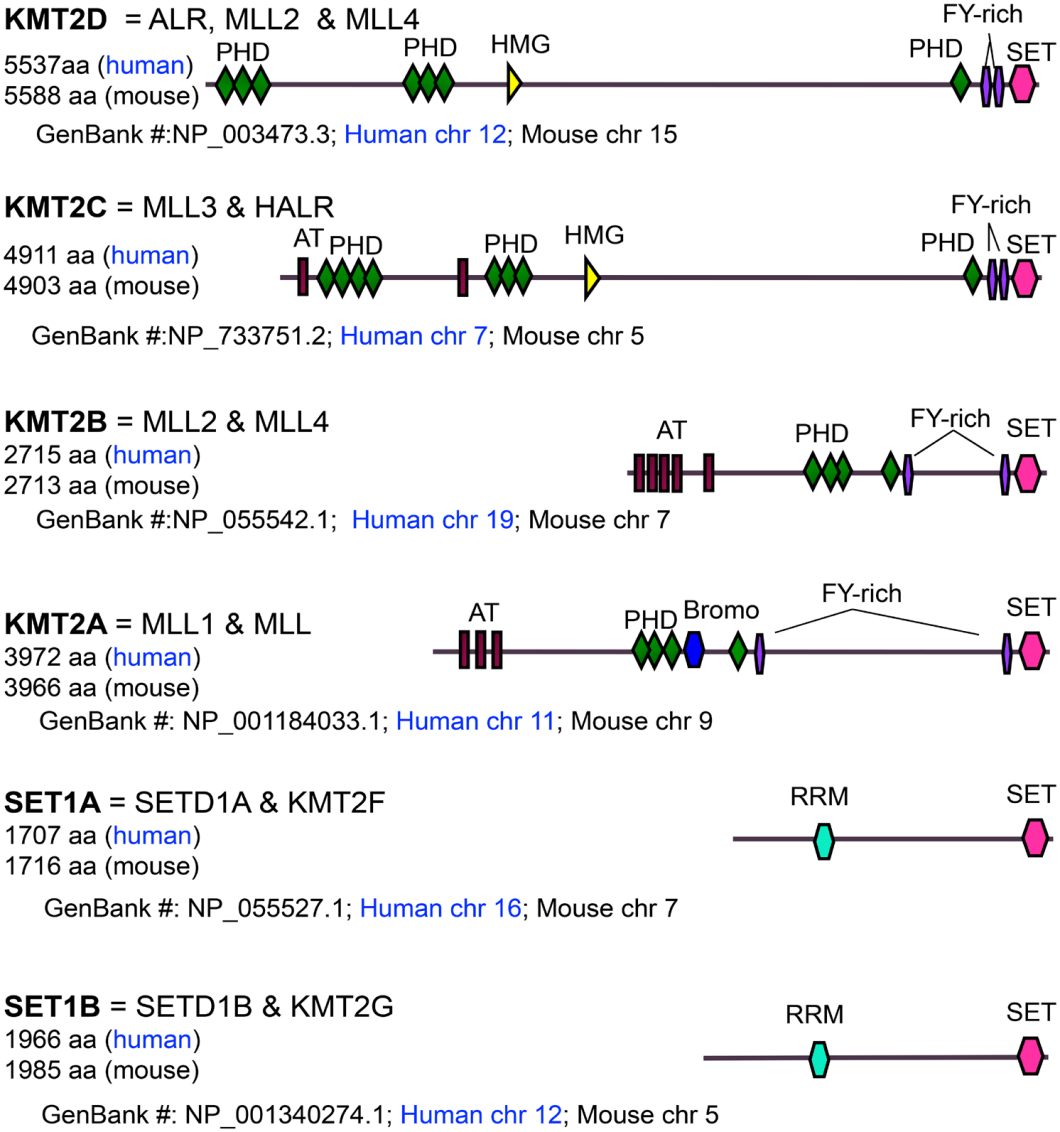
Domain organization, size, and chromosomal location of MLL/SET1 H3K4 methyltransferases. AT: AT-hook DNA binding domain; PHD: Plant Homeodomain; Bromo: Bromodomain; FYR: FY-rich domain; SET: Su(var)3-9, Enhancer of zeste, Trithorax domain; HMG: High Mobility Group domain; RRM: RNA Recognition Motif.

In particular, H3K4 methylation is critical for epigenetic and spatiotemporal activation of gene expression [6–10]. It can be catalyzed by the COMPASS (Complex of Proteins Associated with Set1) family of histone H3K4 methyltransferases, which is conserved from yeast to human and includes SET1A, SET1B, and KMT2A-D (MLL1-4) in mammals (e.g., mouse and human) [11] (Figure 1). Notably, KMT2D (also called MLL4, ALR, and MLL2) is the biggest H3K4 methyltransferase and is frequently mutated in many different types of cancer.

### KMT2D’s nomenclature

KMT2D (human chromosome 12 and mouse chromosome 15) is different from KMT2B (also called MLL2 and MLL4) in both size and chromosomal location, although, confusingly, both of KMT2D and KMT2B have been called MLL2 (Figure 1).

### *KMT2D’s* mutations in cancer

**Figure 2:**
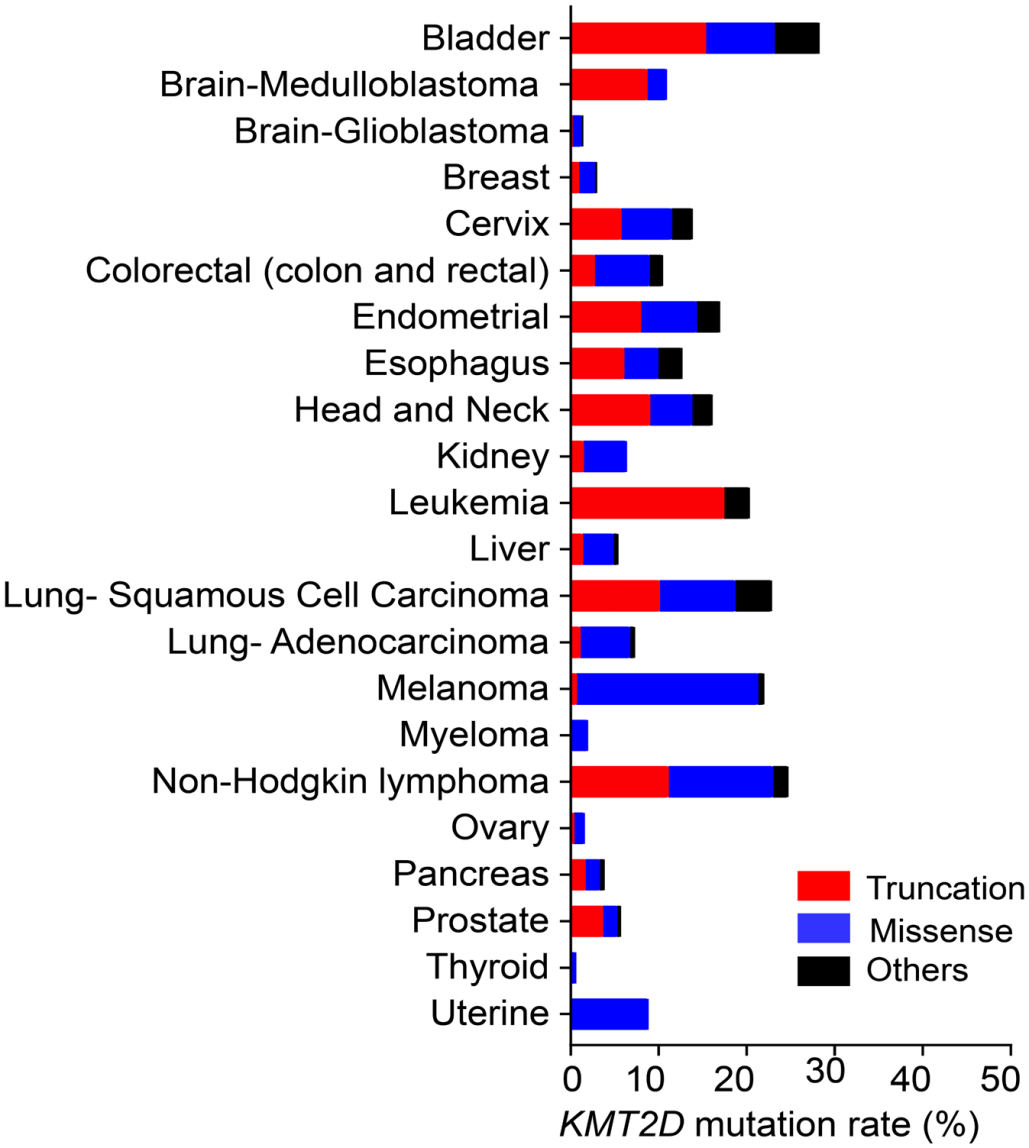
The frequency of truncating, missense, and other (inframe and splice-site) mutations in *KMT2D* in different types of cancer. The mutation frequencies for the cancer types listed, except medulloblastoma (Broad database), leukemia (St Jude database), and Non-Hodgkin lymphoma (Duke database), were obtained from data in TCGA PanCancer Atlas. All data were from the cBioportal database.

The *KMT2D* gene is mutated and deleted in many different types of cancer, including medulloblastoma, melanoma, lymphoma, leukemia, and lung, prostate, renal, bladder, ovarian, pancreatic, esophageal, and gastric cancers [12–28]. *KMT2D* has two frequently occurring genomic alterations: truncation and missense mutations (Figure 2). The frequency of these mutations varies according to cancer type. For example, according to The Cancer Genome Atlas database, melanoma contains much more missense mutations than truncations. In contrast, in medulloblastoma, most *KMT2D* mutations are truncations [29]. Most, if not all, of KMT2D truncations cause catalytic inactivity because the catalytic SET domain (5397aa‒5513aa) is located at the C-terminus of KMT2D (5537aa). Because KMT2D harbors truncating mutations in many types of cancer, KMT2D have been considered a tumor-suppressor in such instances. Of *KMT2D*’s missense mutations in lymphoma, several in the catalytic domain reduce KMT2D’s enzyme activity [30]. However, most missense mutations are located outside the catalytic SET domain in many different types of cancer, including lymphoma. It is unclear whether and how such KMT2D’s missense mutations affect its function, and more research in this area is needed

### KMT2D as a tumor suppressor

Consistent with its loss-of-function mutations, KMT2D can act as a tumor suppressor (Table 1). We have shown that brain-specific loss of *Kmt2d* alone induces spontaneous medulloblastoma in 34.6% of brains in a genetically engineered mouse model (GEMM) [31]. Mechanistically, KMT2D upregulates expression of the tumor suppressor gene *Dnmt3a* whose protein, DNMT3A, downregulates expression of several oncogenic Ras activators to suppress medulloblastoma [31]. In addition, KMT2D activates expression of the tumor suppressor genes *Bcl6* and *Sirt1*, whose proteins repress expression of several oncogenic Notch pathway genes to suppress medulloblastoma [31].

**Table 1:** The tumor-suppressive or pro-tumorigenic functions of KMT2D

Cancer type	Experimental systems for tumorigenesis study	Anti-tumor or pro-tumor	KMT2D-regulated pathways	Ref
Bladder cancer	Cell lines & xenograft model	Anti-tumor	KMT2D → Tumor suppressor genes	[36]
Brain cancer (Medulloblastoma)	GEMM (*Kmt2d*^fl/fl^; *Nestin-Cre*), cerebellar neurospheres & mouse transplantation	Anti-tumor	KMT2D → *Dnmt3a* ┤ Ras activators KMT2D → *Sirt1/Bcl6* ┤ Notch pathway	[31]
	Cell lines	Pro-tumor	N/D	[47]
Breast cancer	Cell lines	Pro-tumor	AKT-mediated phosphorylation of KMT2D ┤ KMT2D activity & ER function	[43]
	Cell lines & xenograft model	Pro-tumor	KMT2D → Genes for tumor growth and cell invasion	[44]
Esophageal cancer	Cell line	Pro-tumor	KMT2D → ? → Epithelial mesenchymal transition	[42]
Gastric cancer	Cell lines & xenograft model	Pro-tumor	KMT2D → ? → Phospho-AKT	[41]
Melanoma	GEMM (*Kmt2d*^fl/fl^ ;*Tyr-CreERT2, Rosa26-rtta; TetO-BRAFV600E; PTEN* ^fl/fl^ *; INK/ARF* ^fl/fl^), cell lines & xenograft model	Anti-tumor	KMT2D → *IGFBP5* ┤ IGF1R signaling → Glycolysis genes	[33]
	PDX models	Pro-tumor	KMT2D → Genes for tumor growth and cell migration	[45]
Leukemia	GEMM (*MLL-AF9*–transduced *Kmt2d*^fl/fl^;*Mx-Cre* cells and transplantation*)*	Pro-tumor	KMT2D → Pro-tumorigenic HOXA9 target genes	[37]
	GEMM (HOXA9/MEIS1–transduced *Kmt2d*^fl/fl^;*ER-Cre* cells and transplantation)	Pro-tumor	KMT2D → Antioxidant response genes	[38]
Lung cancer	GEMM (*Kmt2d*^fl/fl^ ;*K-Ras* ^LSL-G12D^ and intratracheal intubation of Ad5-CMV-Cre), cell lines & xenograft model	Anti-tumor	KMT2D → Circadian rhythm tumor suppressor gene *Per2* ┤ Glycolysis genes	[32]
Lymphoma	GEMM (*Kmt2d*^fl/fl^;*Cγ1-Cre*; VavP-*Bcl2*) & B cells	Anti-tumor	KMT2D → ? ┤ Cell cycle and anti-apoptotic genes	[30]
	GEMM (*Kmt2d*^fl/fl^;*CD19-Cre* and *Kmt2d*^fl/fl^;*CD19-Cre; AID-Tg*) & B cells	Anti-tumor	KMT2D → Tumor suppressor genes	[35]
Pancreatic cancer	Cell lines & xenograft model	Anti-tumor	KMT2D → ? ┤ Glucose transporter SLC2A3 (alias GLUT3)	[34]
	Cell lines	Pro-tumor	KMT2D → Cell cycle genes	[46]
Prostate cancer	Cell lines & xenograft model	Pro-tumor	KMT2D → *KLF4* and *LIFR*	[39]

→: positive regulation; ┤: negative regulation; ?: unknown; GEMM: genetically engineered mouse model; N/D: not determined; PDX: patient-derived xenograft.

We also demonstrated that lung-specific loss of *Kmt2d* significantly promoted K-Ras^G12D^–driven lung tumorigenesis by upregulating tumor-promoting programs [32]. Interestingly, KMT2D activates expression of the tumor suppressor Per2 and thereby downregulates expression of tumor-promoting glycolytic genes in K-Ras^G12D^–driven lung tumors [32]. Similarly, Maitituoheti *et al.* [33] showed that *Kmt2d* loss promoted melanoma in a GEMM and that KMT2D downregulated expression of glycolytic genes by increasing expression of IGFBP5, a tumor suppressor and negative regulator of IGF1R signaling. In a study of pancreatic cancer, Koutsioumpa *et al.* [34] showed that KMT2D levels were downregulated in pancreatic tumors compared to normal pancreatic tissues and that KMT2D decreased glycolysis via downregulation of the glucose transporter SLC2A3 (alias GLUT3) to suppress tumorigenicity of pancreatic cancer cell lines.

Studies have shown that genetic ablation of *Kmt2d* or KMT2D knockdown accelerated the oncogene (Bcl2)-driven lymphomagenesis of B cells in mice [30, 35]. In addition, *Kmt2d* deletion in CD19+ early B cells induced spontaneous lymphoma in 58% of mice [35]. KMT2D suppressed lymphoma by activating pro-apoptotic genes while repressing genes associated with cell growth and survival pathways, such as cell cycle and anti-apoptotic genes [30, 35]. A study of bladder cancer showed that KMT2D was required to maintain tumor suppressor genes and that it impeded the tumorigenicity and invasiveness of bladder cancer cells [36].

### KMT2D’s pro-tumorigenic function

In contrast to its tumor-suppressive function, KMT2D appears to activate tumor-promoting pathways in certain contexts or tissue-specific manners (Table 1). Santos *et al.* [37] have shown that KMT2D upregulates gene expression programs for antioxidant responses to protect against reactive oxygen species and DNA damage. Thus, it is indispensable for leukemogenesis induced by the fusion oncogene MLL–AF9 *in vivo*. Another leukemia study showed that KMT2D occupied and activated pro-tumorigenic HOXA9 target genes and that KMT2D was required for leukemogenesis mediated by co-expression of *HOXA9* and *MEIS1* *in vivo* [38].

In addition, Lv *et al.* [39] have shown that KMT2D knockdown reduced the PI3K/AKT pathway to decrease the proliferation and invasion of prostate cancer cell lines. This study also showed that KMT2D activated expression of KLF4 (a transcription factor promoting cell invasion and LIFR expression) and LIFR (a receptor activating the PI3K/AKT pathway). Similarly, studies using gastric cancer cell lines showed that KMT2D knockdown reduced phospho-AKT signals and cell proliferation [40, 41]. In esophageal squamous cell carcinoma cells, *KMT2D* knockout inhibited cell proliferation and migration and reduced epithelial mesenchymal transition [42].

A study using estrogen receptor (ER)-positive breast cancer cell lines showed that KMT2D knockdown reduced cell viability and increased the sensitivity of xenograft tumors to a PI3Kα inhibitor. In addition, KMT2D played a critical role in recruiting ER to ER target genes and activating such genes [43]. In a breast cancer study, we showed that KMT2D knockdown inhibited cell proliferation and invasion of triple–negative breast cancer cell lines and that KMT2D and KMT2D-interacting histone demethylase UTX co-activated gene expression programs for cell proliferation and invasion [44].

In patient-derived xenograft (PDX) melanoma models, KMT2D was required for the growth and invasion of N-RAS-mutated melanoma and positively regulated genes for tumor growth and cell migration [45]. This finding appears to be in disagreement with the above-described melanoma study by Maitituoheti *et al.* [33], which showed a tumor-suppressive role for KMT2D. Dawkins *et al.* [46] reported that high KMT2D levels correlated with worse prognosis in patients with pancreatic cancer and that KMT2D positively regulated cell cycle genes in several pancreatic cancer cell lines. This conclusion appears to be discordant with the aforementioned pancreatic study by Koutsioumpa *et al.* [34], although these two studies used different pancreatic cancer cell lines. Guo *et al*. [47] showed that KMT2D deficiency attenuated the proliferative and migratory ability of medulloblastoma cell lines and others. This is also in discrepancy with ours, which showed medulloblastoma-suppressive function of KMT2D using a GEMM [31]. In these studies, although the same types of cancer were studied, KMT2D could be anti-tumorigenic or pro-tumorigenic, depending on the model systems used (cell lines, PDX models, and GEMM). These discrepancies are further discussed in the following section.

### KMT2D: tumor suppressor vs pro-tumorigenic functions

An important question is how KMT2D can have tumor-suppressive or pro-tumorigenic function for a specific tissue, depending on whether the model system is a GEMM, a human cancer cell line, or a PDX model? GEMM models can harbor tumors that result from a few defined genetic alterations, whereas human cancer cell lines and PDX models often harbor multiple mutations. Such mutations may render a cancer cell line or PDX tumor dependent on KMT2D for survival. In such cases, KMT2D deficiency can attenuate the proliferation of affected cell lines or the growth of PDX tumors, even if KMT2D is a tumor suppressor. This point can be supported by the notion that simultaneous inactivation of two tumor suppressor genes, such as BRCA1 and Poly(ADP-ribose) polymerase, leads to synthetic lethality (cell death) [48]. Another point is that human cell lines, xenograft tumors, and PDX tumors are usually grown in immunodeficient conditions. In contrast, the autochthonous growth of tumors in GEMMs occurs in native microenvironments. These microenvironments may involve intact immune systems that would better support the growth of KMT2D-deficient tumors than that of KMT2D-wild type (WT) tumors. Supporting this point, it was shown that KMT2D-deficient syngeneic tumors were more sensitive to immunotherapy than were KMT2D-WT tumors [49] and that immune response pathways were more enriched in KMT2D-deficient melanoma than in KMT2D-WT melanoma [33].

Another critical question is how KMT2D can act as a tumor suppressor or a pro-tumorigenic factor in a tissue-dependent manner. One possibility is that tissue-specific transcription factors direct the KMT2D complex to a unique set of tumor-promoting genes or tumor suppressor genes in a tissue-dependent manner. For example, KMT2D associates with the tumorigenic factor HOXA9 during leukemogenesis [38]. Relevant to this question, the KMT2D complex can associate with lineage-specific DNA-binding transcription factors. Studies have shown that p63 interacts with KMT2D, which occupies and establishes enhancers for p63 target genes in keratinocytes [50] and that MyoD associates with the KMT2D complex to induce myocyte differentiation [51]. In addition, PPARγ and C/EBP interact with the KMT2D complex to induce adipocyte differentiation [51]. Another possibility—not mutually exclusive with the above possibility—is that post-translational modifications may regulate KMT2D function in a tissue-dependent manner.

### KMT2D-catalyzed H3K4 methylation in cancer cells

KMT2D was initially identified as ALR in a multi-protein complex containing activating signal cointegrator 2 (ASC2) and other proteins [52]. Later, we showed that the KMT2D complex included the H3K27 demethylase UTX, PTIP, and other subunits (e.g., RBBP5 and WDR5) [53]. In parallel, other groups found these subunits in the KMT2D complex [54, 55]. We demonstrated that immunoprecipitates containing full-length or minimal versions of KMT2D can catalyze H3K4me1, H3K4me2, and H3K4me3 *in vitro* [53, 56]. In addition, recombinant core subunits of KMT2D (WDR5, ASH2L, RBBP5 and DPY30) conferred H3K4 tri-methyltransferase activity on a recombinant KMT2D fragment containing the catalytic domain SET, which has a monomethyltransferase H3K4 activity along with a much weaker dimethyltransferase activity [57]. Therefore, KMT2D is capable of catalyzing H3K4m1, H3K4me2, and H3K4me3.

Interestingly, it has been shown that KMT2D acts as a H3K4 mono-methyltransferase or a H3K4 mono- and dimethyltransferase in mammalian cells [51, 58, 59]. In these studies, the effects of *KMT2D* loss on H3K4 methylation were examined using mammalian cells with *MLL3*-null states (e.g., human HCT116 colon cancer cells, mouse brown adipocytes, and mouse embryonic stem cells) because KMT2D and MLL3 (homologues of the *Drosophila* H3K4 methyltransferase Trr) are closely related to each other. Our study also showed that KMT2D loss in K-Ras^G12D^–driven lung tumors reduced global H3K4me1 levels while not having an obvious effect on global H3K4me3 levels [32].

In addition to KMT2D-catalyzed mono- and dimethylation of H3K4, KMT2D-mediated H3K4 trimethylation has been reported in other cell lines and animal models, consistent with the *in vitro* activity of KMT2D. We have shown that loss of the *Kmt2d* gene in the mouse brain reduced H3K4me1 and H3K4me3 levels in many genes during medulloblastoma genesis. We have also reported that KMT2D activates gene expression by upregulating H3K4me3 at gene promoters in breast cancer cells and in the neuron-committed human embryonal carcinoma cell line NT2/D1 [44, 56]. Consistent with this, *Kmt2d* loss in mouse B cells during lymphomagenesis decreases global H3K4me1, H3K4me2, and H3K4me3 levels [30].

These studies indicate that KMT2D monomethylates H3K4 and can trimethylate H3K4 in a cell-type-specific manner. Although it is not clear how KMT2D–catalyzed H3K4 trimethylation is regulated in a cell-type-specific manner, one possibility is that the distinct composition or expression patterns of the core subunits WDR5, ASH2L, RBBP5, and DPY30 in different cell types play a role in KMT2D-mediated H3K4 trimethylation. Future studies may determine the detailed mechanism underlying KMT2D-mediated H3K4 trimethylation.

### KMT2D-mediated regulation of enhancers and super-enhancers

Enhancers are a critical gene-activating signature that spatiotemporally enhances gene expression by interacting with and activating the promoters. Monomethyl H3K4 (H3K4me1) and acetyl H3K27 (H3K27ac) decorate the enhancers, which are co-occupied by transcriptional activators and co-activators (e.g., the histone acetyltransferases p300 and CBP) [60, 61]. H3K27ac signifies active states of the enhancers and can be generated by p300 and CBP [62]. Large clusters of enhancers, called super-enhancers, highly activate gene expression and determine cell fate and cell identity [63–65]. In addition, super-enhancers can activate expression of oncogenes [66] and be associated with specific cancer subtypes [67].

**Figure 3:**
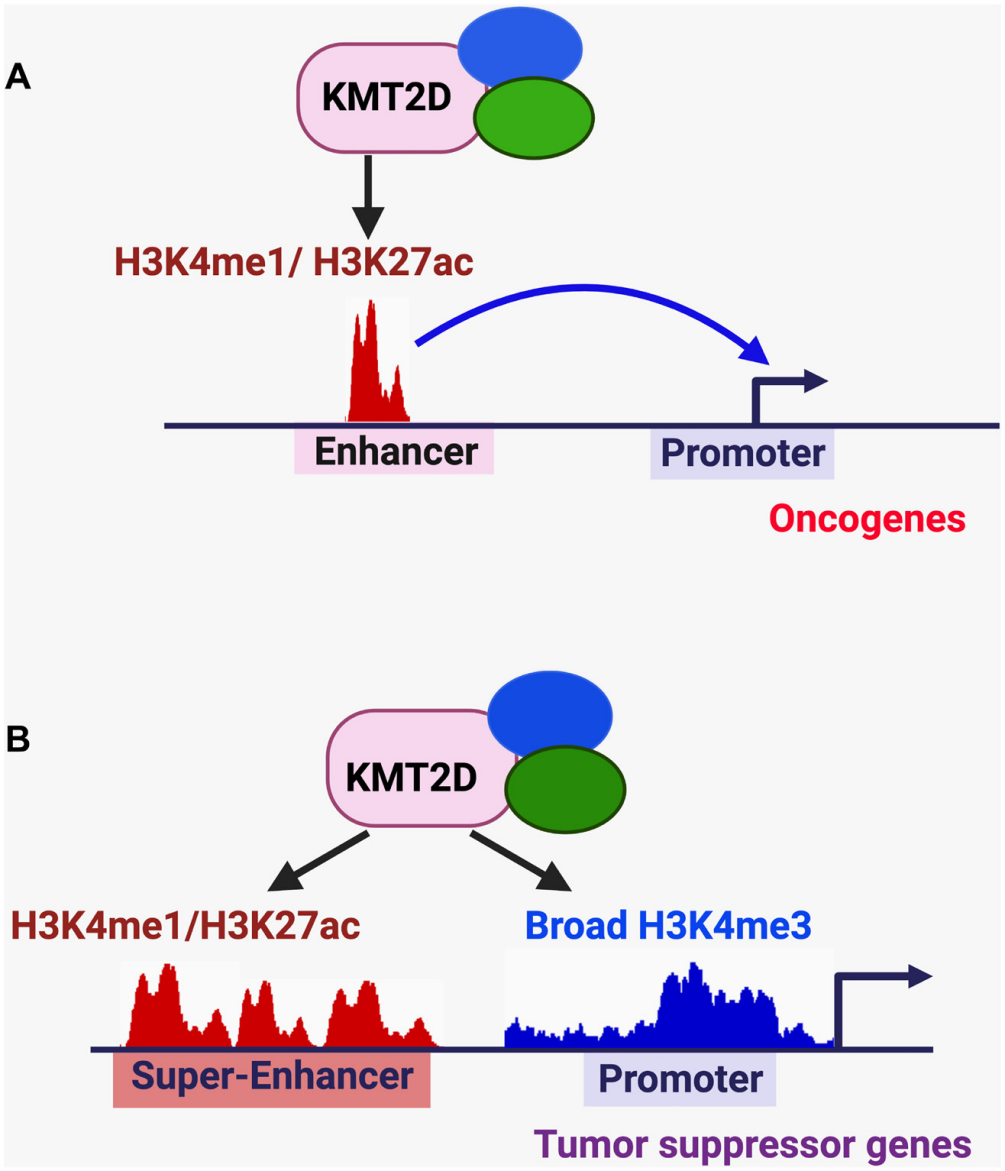
KMT2D-mediated regulation of enhancers, super-enhancers, and broad H3K4me3 signature in cancer. (**A**) KMT2D-mediated activation of pro-tumorigenic enhancers. (**B**) KMT2D-mediated activation of tumor-suppressive super-enhancers/enhancers and broad H3K4me3 peaks.

It has been reported that KMT2D activates pro-tumorigenic enhancers in different types of cancer (Figure 3A). For instance, in N-RAS-mutated melanoma, KMT2D activated enhancers for the cell migration-associated genes *MFGE8* and *RPL39L* [45]. In ER-positive breast cancer cells, SGK1-mediated phosphorylation of KMT2D attenuated the activity and chromatin occupancy of KMT2D to downregulate the enhancer signal H3K4me1 at oncogenes (e.g., *MYC* and *cFOS*) [68].

In contrast to oncogenic super-enhancers, some super-enhancers are associated with tumor suppressor genes (Figure 3B). We have shown that KMT2D-activated tumor-suppressive super-enhancers interact with the promoters of the DNA methyltransferase gene *Dnmt3a* and the medulloblastoma suppressor gene *Bcl6* and upregulate these genes to suppress medulloblastoma genesis [31]. We have also shown that expression of the circadian transcription–repressive tumor suppressor Per2 is positively regulated by a KMT2D–activated, tumor-suppressive super-enhancer to attenuate lung tumorigenesis [32]. Moreover, KMT2D positively regulates the enhancer of the tumor suppressor gene *IGFBP5* for melanoma suppression [33] and the enhancers of several tumor suppressor genes (e.g., *Socs3*, *Asxl1* and *Arid1A*) for lymphoma suppression [35]. Therefore, KMT2D plays a critical role in activating tumor-suppressive enhancers and super-enhancers. Of note, KMT2D is also required for the positive regulation of enhancers and super-enhancers in other cellular processes, such as the exit from naive pluripotency [69], embryonic stem cell differentiation [59], and adipogenesis [70].

### KMT2D-mediated regulation of the tumor-suppressive, broad H3K4me3 signature

H3K4me3 is generally located in gene-regulatory regions spanning the promoters and the transcription start sites [10, 71]. This mark is positively associated with the transcription frequency of active genes [7, 8], although the co-occupancy of H3K4me3 and the repressive mark H3K27me3 (called bivalent domains) represents poised (activatable but repressed) states [72, 73]. Remarkably, H3K4me3 occupies up to 79% of all protein-coding gene promoters in human cells, such as human embryonic stem cells [74–76]. Thus, it is one of most abundant and important histone marks.

Sharp and broad H3K4me3 peaks are two major types of H3K4me3 signatures. Sharp H3K4me3 peaks are a high and narrow signature that is enriched in the approximately 1-kb symmetric regions around the transcription start sites [77]. Broad H3K4me3 peaks (also called H3K4me3 breadth) [78] cover at least from -500 bp to +3,500 bp and signify highly active genes. Broad H3K4me3 peaks are associated with cell identity and tumor suppression, are reduced in cancer cells, and inversely correlate with DNA methylation [77, 78]. We have previously demonstrated that homozygous *kmt2d* loss in the brain downregulates broad H3K4me3 peaks to decrease expression of tumor suppressor genes (Figure 3B) [31]. In contrast, we showed that homozygous *kmt2d* loss in K-Ras^G12D^–driven lung tumors did not obviously change the average size of H3K4me3 peaks [32]. These studies suggest that KMT2D positively regulates broad H3K4me3 peaks in a tissue-dependent manner.

### KMT2D-regulating pathways

**Figure 4:**
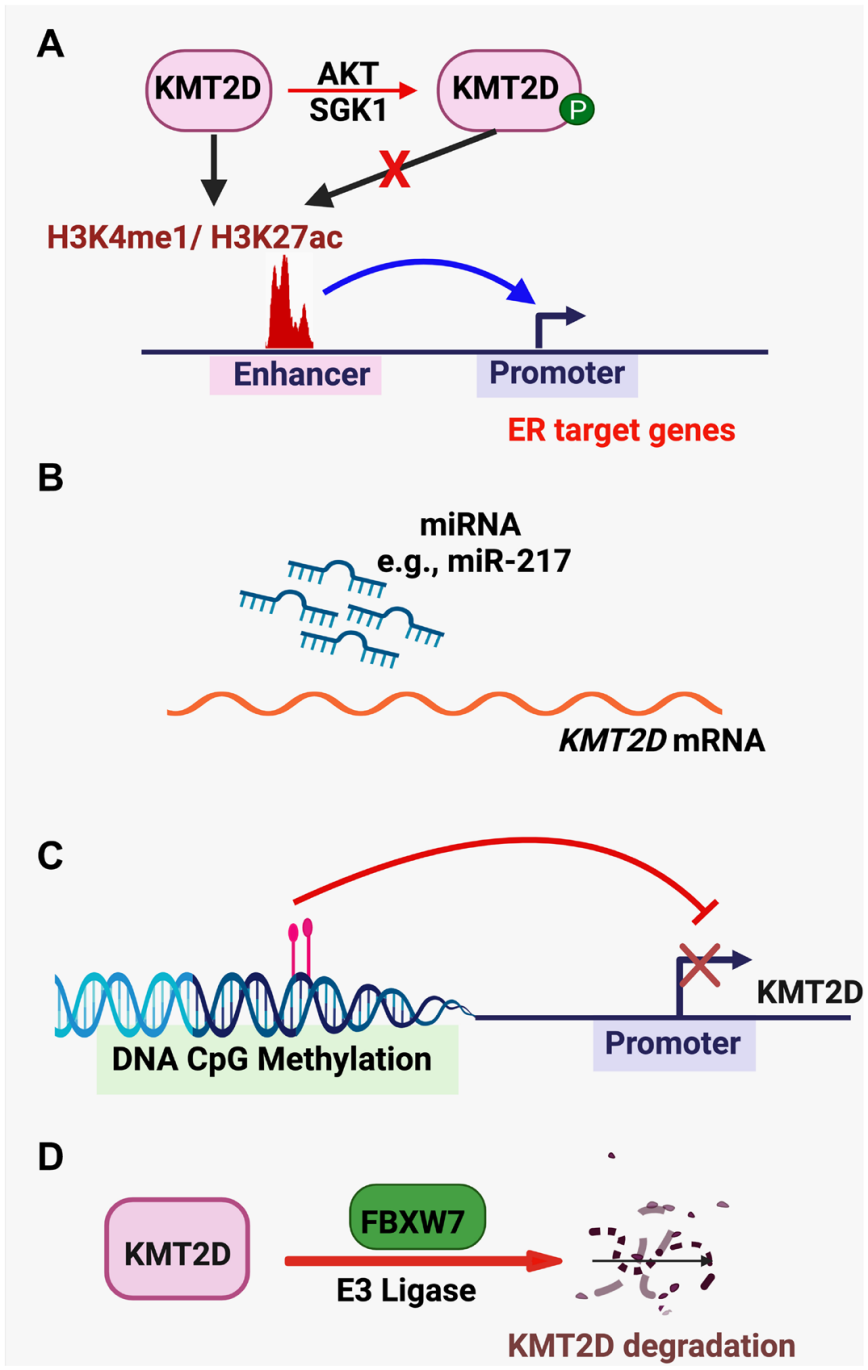
KMT2D-regulating pathways. (**A**) The kinases AKT and SGK1 phosphorylate KMT2D to reduce its methyltransferase activity. (**B**) miRNAs (e.g., miR-217) targets *KMT2D* mRNA to reduce *KMT2D* expression. (**C**) *KMT2D* expression is repressed by DNA CpG methylation. (**D**) The E3 ligase FBXW7 targets KMT2D for protein degradation.

KMT2D function is regulated in several different mechanisms (Figure 4A–4D). The kinases AKT and SGK1 interact with and phosphorylate KMT2D to reduce its methyltransferase activity, which is important for ER’s tumor-promoting function in ER-positive breast cancer cell lines [43, 68]. It was reported that miR-217 promoted the proliferation and migration of bladder cancer cells by targeting KMT2D and that miR-217 levels were negatively correlated with KMT2D expression in bladder cancer samples [79]. KMT2D expression levels can be downregulated by DNA CpG methylation in pancreatic cancer cells [34]. Saffie *et al.* [80] have shown that the E3 ligase FBXW7 interacts with KMT2D and promotes KMT2D degradation to increase the proliferation of diffuse large B-cell lymphoma cells.

### The clonality and therapeutic implications of KMT2D’s mutations

If gene mutations occur as early events and are present in all cancer cells during tumorigenesis, such mutations are clonal (truncal). The clonality of mutations is important because subclonal (branched) alterations may exist in only a subset of cancer cells and thus limit treatment efficacy if tumors bearing such mutations are treated [81]. If mutations of a gene are clonal, the resultant gene products or their downstream effectors can be candidate actionable targets for cancer treatment. A study has shown that the majority of *KMT2D* mutations (e.g., 6 out of 9 mutations examined) in non-small cell lung cancer were clonal [82]. Moreover, all KMT2D mutations examined in esophageal tumors was clonal [83]. These studies would justify pharmacological targeting of oncogenic pathways altered by KMT2D deficiency (KMT2D truncation or loss) for the treatment of non-small cell lung tumors and esophageal tumors bearing *KMT2D* mutations. For other types of cancer, the clonality of KMT2D mutations remains to be investigated.

### Therapeutic opportunities for the treatment of KMT2D-deficient tumors

Consistent with our finding that *Kmt2d* loss increases glycolysis in lung tumorigenesis, [32], we showed that pharmacological inhibition of glycolysis using 2-deoxy-D-glucose impeded the proliferation of human lung cancer cell lines bearing KMT2D truncations to a greater extent than that of KMT2D-WT lung cancer cell lines and selectively inhibited the tumorigenicity of KMT2D-deficient lung cancer cells in mice [32]. Similarly, pharmacological inhibition of the glycolysis pathway using 2-deoxy-D-glucose, POMHEX (an enolase1 inhibitor), and lonidamine (a hexokinase inhibitor) selectively impeded the proliferation of KMT2D-mutant melanoma cells compared with that of KMT2D WT melanoma cells [33]. However, the OXPHOS inhibitor IACS-010759 did not have a significant selective inhibitory effect on the proliferation of KMT2D-deficient cancer cells compared to that of KMT2D-WT cancer cells [32, 33].

Interestingly, pharmacological inhibition of the KDM5 family of H3K4 demethylases diminished tumorigenic growth of KMT2D-mutant lymphoma cells, as certain KDM5 family members can antagonize the function and methylation activity of KMT2D [84]. Other study has shown that KMT2D deficiency sensitizes different types of syngeneic tumors (including lung tumor, bladder tumor, breast tumor, and melanoma) to anti-PD1 [49], suggesting that anti-PD1 may have a stronger inhibitory effect on KMT2D-mutant tumors than on KMT2D-WT tumors. In addition, KMT2D deficiency augments the tumor-inhibitory effect of the curcumin analog L48H37 on pancreatic cancer cells [85] and increases the sensitivity of other cancer cells to chemotherapeutic drugs, such as doxorubicin, carboplatin, and the nucleotide analog Fluorouracil [46, 86]. Furthermore, KMT2D knockdown increases the sensitivity of head and neck squamous cell carcinoma cells to Aurora kinase inhibitors [87]. These studies indicate therapeutic opportunities for the treatment of KMT2D-deficient tumors.

### Other aspects of KMT2D studies

As mentioned above, KMT2D has been described as either a tumorigenic factor or a tumor suppressor on the basis of results from the use of human cancer cell lines and their xenograft tumor models. Such results have also provided mechanistic insights into how KMT2D regulates tumorigenicity. However, because experiments using human cancer cell lines do not use native tumor microenvironments and human cancer cell lines often harbor multiple uncharacterized mutations, future studies using genetically defined mouse models (e.g., GEMMs) are desirable. This approach may allow researchers to assess whether results from cancer cell line experiments would be reproduced using GEMM experiments.

Histone modifiers often posttranslationally modify non-histone substrates relevant to tumorigenesis. For instance, the histone lysine methyltransferase SMYD3 methylates the kinase MAP3K2 to upregulate MAP kinase signaling and accelerate K-Ras^G12D^-driven tumorigenesis [88]. It is possible that KMT2D-mediated methylation alters the functions of certain oncogenic factors or tumor suppressors to regulate tumorigenesis. Therefore, in future studies, it would be interesting to determine whether KMT2D regulates oncogenic factors or tumor suppressors via methylation.

The use of standard chemotherapy medicines, small molecular inhibitors, and immune checkpoint inhibitors has provided survival benefits for cancer patients. To improve cancer treatment, drug-combination therapies have frequently been used. Thus, it would be useful to study drug-combination approaches as therapeutic strategies of the treatment of KMT2D-deficient tumors. Recently, Tazemetostat, which inhibits the histone methyltransferase EZH2, was approved as the first epigenetic drug of solid tumors (epithelioid sarcomas and follicular lymphoma). Other epigenetic inhibitors are in clinical trials for the treatment of different cancers [89]. Thus, targeting altered epigenetic modifiers and their downstream effectors is a viable and promising option for cancer treatment.
